# Application of Nonfungible Tokens to Health Care. Comment on “Blockchain Technology Projects to Provide Telemedical Services: Systematic Review”

**DOI:** 10.2196/34276

**Published:** 2022-05-30

**Authors:** John Gambril, Carter Boyd, Jamal Egbaria

**Affiliations:** 1 Department of Internal Medicine Ohio State University Wexner Medical Center Columbus, OH United States; 2 Department of Pediatrics Nationwide Children's Hospital Columbus, OH United States; 3 Hansjörg Wyss Department of Plastic Surgery New York University Langone Health New York, NY United States; 4 Department of Anesthesiology and Perioperative Medicine University of Alabama at Birmingham School of Medicine Birmingham, AL United States

**Keywords:** telemedicine, distributed ledger, health information exchange, blockchain, cryptocurrency, nonfungible token, non-fungible token, medical education, internet, finance

In their systematic review, Koshechkin and colleagues [1] thoughtfully explored and summarized the potential applications of blockchain technology to solve unique challenges in health care. Blockchain technology is a highly secure but transparent method of tracing digital transactions of assets or information through a decentralized, immutable ledger [1]. Thanks to the expansion of cryptocurrency—digital currency traded through blockchain networks—blockchain has become a household term. Blockchain technology has been applied to numerous industries, and many possibilities exist for application within health care [2].

In their review, Koshechkin and colleagues [1] identified 18 studies addressing blockchain solutions for various health care challenges. These included medical data access, medical services processing, diagnostic support, payment transactions, and fundraising, among others. This review offers an excellent synopsis of ongoing blockchain projects in health care accompanied by discussion of future directions.

One novel entity that utilizes blockchain technology not identified in this paper is the nonfungible token (NFT). NFTs are similar to cryptocurrency, such as Bitcoin, in that they are digital tokens existing within a blockchain that can be bought and sold. NFTs, however, are distinct tokens. While 1 Bitcoin is always equal to another Bitcoin, each NFT is singular and unique (ie, nonfungible) [3]. Interestingly, although NFTs are digital, they can, and often do, represent physical items.

Regarding health care, NFTs have been suggested as a means to streamline and simplify blood [4] and stem cell [5] product supply chains. While the full discussion of NFT potential in health care warrants a lengthier article, we will touch on the simplest application: capital.

NFTs represent an untapped method of fundraising and revenue generation. They allow the monetization of unique items and content, digital or physical, new or old. NFTs offer the added benefits of transaction tracing, verifiable authenticity, and shared ownership of physical objects (analogous to stockholders of a company). One can imagine NFT auctions of historic medical equipment or journal articles, recorded lectures from renowned experts, and even naming rights to a lecture series. Much like baseball cards or stamps, medicine could find its own niche in collecting NFTs.

NFTs also open an avenue for monetization of original creations such as reflective art and literature or unique medical education content. Medical education resources are constantly evolving, and many are free to learners (tweetorials, podcasts, YouTube channels, etc). If collectors agree to maintain open access after purchase, leveraging this content as NFTs could represent a new market to raise funds for scholarships, research, advocacy, or public health projects. Many will argue that buyers are unlikely to permit open access to their NFT. However, as already seen in other NFT markets, because NFTs are unique and traceable digital tokens, ownership can still be boasted despite public access [3].

As illustrated by Koshechkin and colleagues’ [1] review, blockchain technology is an exciting entity offering much potential for the advancement of health care. NFTs are yet another example of blockchain technology with innumerable possibilities and potential in health care.

## Abbreviations

NFT: nonfungible token
